# Supplementary material for: Complexome profiling of the Chlamydomonas psb28 mutant reveals TEF5 as an early PSII assembly factor
Source: Plant Cell. 2025 Mar 17;37(6):koaf055. doi: 10.1093/plcell/koaf055 (PMC12164590; doi:10.1093/plcell/koaf055)
Supplement: koaf055_Supplementary_Data [file koaf055_supplementary_data.zip › psb28_interactive.zip!/plots/intensity/qualityPlots/peptides/CGL102_Cre02.g142146.t1.1.html]

### Cre02.g142146.t1.1

Trivial name: CGL102  
SeqCoverage [median]: 39.80  
Mass: 40.86 kDa  
  
 

| Sequence | MissedCleavage | Mass [Da] | wtIntensity | muIntensity | Score |
| --- | --- | --- | --- | --- | --- |
| ALDDGYDVR | 0 | 1022.47 | 16069.80 | 34038.14 | 156.01 |
| EVGASADR | 0 | 803.38 | 0.00 | 3244.58 | 122.13 |
| EVGASADRTNFYV | 1 | 1427.67 | 771.15 | 392.72 | 154.24 |
| FLEASSLDYTTFR | 0 | 1548.75 | 1106.62 | 8017.74 | 157.68 |
| KTLPLSGPK | 1 | 939.58 | 14954.43 | 29085.28 | 133.99 |
| KVDWEGK | 1 | 860.44 | 1953.70 | 949.01 | 102.00 |
| LKEVGASADRTNFYV | 2 | 1668.85 | 4503.14 | 9290.22 | 77.73 |
| MTMAALR | 0 | 792.40 | 5829.33 | 11035.44 | 128.48 |
| MTMAALRGDK | 1 | 1092.54 | 23444.61 | 31798.55 | 93.56 |
| MTMAALRGDKTSR | 2 | 1436.72 | 979.45 | 1513.37 | 96.82 |
| NSILVVGATGTLGR | 0 | 1356.77 | 9607.32 | 80714.45 | 201.38 |
| PNPADFLR | 0 | 928.48 | 0.00 | 3438.43 | 73.26 |
| PRPNPADFLR | 1 | 1181.63 | 0.00 | 4091.25 | 77.38 |
| RALDDGYDVR | 1 | 1178.57 | 379.15 | 1372.90 | 54.47 |
| SMQWAADAADR | 0 | 1220.52 | 1903.71 | 1680.90 | 111.06 |
| SVWGTNDETR | 0 | 1163.52 | 2415.71 | 10844.24 | 96.95 |
| SVWGTNDETRTAYLDSQDVAK | 1 | 2355.10 | 1379.65 | 3816.61 | 62.62 |
| TAYLDSQDVAK | 0 | 1209.59 | 48483.64 | 87695.68 | 231.38 |
| TLPLSGPK | 0 | 811.48 | 7984.78 | 17741.38 | 71.34 |
| VALIQSAQAMGIQR | 0 | 1484.81 | 16011.90 | 58500.05 | 223.03 |
| VTTVPTWLLK | 0 | 1156.69 | 2668.16 | 6337.11 | 92.44 |
| VTTVPTWLLKR | 1 | 1312.79 | 7814.56 | 22724.55 | 113.69 |
|  |  |  | 168260.8 | 428322.6 |  |
